# Rice disease detection method based on multi-scale dynamic feature fusion

**DOI:** 10.3389/fpls.2025.1543986

**Published:** 2025-05-13

**Authors:** Qian Fan, Runhao Chen, Bin Li

**Affiliations:** School of Artificial Intelligence, Yangzhou University, Yangzhou, China

**Keywords:** inner-WIoU, rice disease detection, multi-scale feature fusion, flexiC3k2Net, deep learning

## Abstract

In order to enhance the accuracy of rice leaf disease detection in complex farmland environments, and facilitate the deployment of the deep learning model onto mobile terminals for rapid real-time inference, this paper introduces a disease detection network titled YOLOv11 Multi-scale Dynamic Feature Fusion for Rice Disease Detection (YOLOv11-MSDFF-RiceD). The model adopts the concept of ParameterNet to design the FlexiC3k2Net module, which replaces the neck feature extraction network, thereby bolstering the model's feature learning capabilities without significantly increasing computational complexity. Additionally, an efficient multi-scale feature fusion module (EMFFM) is devised, improving both the computational efficiency and feature extraction capabilities of the model, while simultaneously reducing the number of parameters and memory footprint. The bounding box regression loss function, inner-WIoU, utilizes auxiliary bounding boxes and scale factors. Finally, the Dependency Graph (DepGraph) pruning model is employed to minimize the model's size, computational load, and parameter count, with only a moderate sacrifice in accuracy. Compared to the original YOLOv11n model, the optimized model achieves reductions in computational complexity, parameter scale, and memory usage by 50.7%, 49.6%, and 36.9%, respectively, with only a 1.7% improvement in mAP@0.5:0.9. These optimizations enable efficient deployment on resource-constrained mobile devices, making the model highly suitable for real-time disease detection in practical agricultural scenarios where hardware limitations are critical. Consequently, the improved model proposed in this paper effectively detects rice disease targets in complex environments, providing theoretical and technical support for the deployment and application of mobile terminal detection devices, such as rice disease detectors, in practical scenarios.

## 1 Introduction

Rice is a key crop for national food security, and its growth status is extremely sensitive to diseases. The occurrence of diseases is usually related to improper agricultural technology practice, inappropriate variety selection and non-standard use of pesticides, which may lead to the aggravation of diseases. According to the forecast of the National Agricultural Technology Extension Service Center, based on the comprehensive analysis of the base of rice diseases (Muehe et al., 2019) and insect pests, cultivation management methods, variety layout (Singh et al., 2021) and climatic conditions (Hajjar et al., 2023), the occurrence trend of rice diseases in China is expected to be more serious in 2024, and the affected area may reach 390 million acre (Hunan Agriculture, 2024). The increase in the diversity of rice diseases, the acceleration of transmission, and the increase in the difficulty of prevention and control have made the early identification and effective prevention of diseases particularly critical in modern agricultural production. Therefore, the implementation of large-scale and intelligent rice disease management strategies is of great significance for controlling disease spread and ensuring food production.

Compared with traditional computer vision technology, deep learning technology has shown excellent generalization performance in the field of image analysis due to its remarkable ability in feature extraction. This technology has been introduced into the research of agricultural plant disease recognition, and with the rapid development of deep learning technology, its application in the field of target detection has also received extensive attention. Target detection technology is mainly divided into two categories: two-stage method and one-stage method. The Two-stage method decomposes the object detection task into two independent stages: first, the region proposal network (RPN) is used to generate candidate regions; secondly, these candidate regions are classified and accurately located. Representative algorithms include Mask R-CNN (Dorrer and Alekhin, 2021) and Faster R-CNN (Ren et al., 2017). The advantage of this kind of method is that it has lower error recognition rate and missed detection rate, and can achieve higher detection accuracy. However, since it contains two separate calculation steps, the two-stage method has certain limitations in processing speed and is difficult to meet the needs of real-time detection. In order to solve this problem, the one-stage method was developed. Representatives of such methods include YOLO (Wang et al., 2024; Wang et al., 2024b) (You Only Look Once) series and SSD (Zeng et al., 2022) (Single Shot MultiBox Detector). Unlike the two-stage method, the one-stage method merges the recognition and localization process into a single stage. By dividing the image into multiple grids and predicting the category and location of the target simultaneously on each grid, fast target detection is achieved. The advantage of this method is its fast recognition speed, which can meet the needs of real-time detection. In addition, due to the small number of model parameters and high computational efficiency, the one-stage method is also easier to be deployed to mobile devices and embedded systems to achieve edge computing. It is worth noting that with the continuous optimization of the algorithm, the one-stage method has also achieved a significant improvement in accuracy. In some cases, the one-stage method can even surpass two-stage method to achieve a fairly high level of detection. This shows that the one-stage method has broad application prospects in the field of target detection. (Zhan et al., 2024) based on the improved target detection model BHC-YOLOV8 of YOLOv8, which is specifically used to detect tea diseases and defects in real scenes. By introducing the dynamic sparse attention mechanism BiFormer, Haar wavelet improved downsampling module and new feature fusion network, the model has improved in terms of computational complexity, confidence and mAP0.5, which effectively improves the accuracy and efficiency of tea disease and defect detection. (Wang et al., 2024) proposed a lightweight apple leaf disease detection method called LCGSC-YOLO. This method combines LCNet backbone network, GSConv and VOVGSCSP modules, and coordinate attention mechanism to achieve high-efficiency and high-precision disease detection under the YOLO framework. It has low model parameters and computational complexity, and high detection speed, which is suitable for deployment on embedded devices. (Xie et al., 2024) proposed a detection method called YOLO-Sizelect, which realized the accurate and rapid detection of ginseng fruit in natural agricultural environment by integrating C3f-RN feature extraction module and model compression technology. (Liu et al., 2024) developed an early detection method for pine wilt disease based on UAV remote sensing, hyperspectral image reconstruction and support vector machine (SVM) classification. In particular, a new hyperspectral reconstruction network DW3D was proposed to improve the detection efficiency and real-time performance. A lightweight recognition model of plant diseases and insect pests (PDLM-TK) based on tensor features and knowledge distillation was proposed by (Zhang et al., 2024) The model improves the diagnostic efficiency and accuracy of plant diseases and insect pests by constructing a lightweight residual block based on spatial tensor (LRBST), a branch network fusion graph convolution feature (BNF-GC) and a model training strategy based on knowledge distillation (MTS-KD).

In practical agricultural scenarios, especially in resource-constrained environments like mobile terminals, the efficient utilization of computational resources is of great significance. Models with lower computational complexity and smaller memory footprint can be deployed more easily on these devices, enabling real-time and on-site disease detection. Therefore, in addition to recognition accuracy, the resource conservation capability of a disease detection model is equally important for its practical application. Our proposed YOLOv11-MSDFF-RiceD model focuses on achieving this balance by optimizing the model structure to reduce computational load and memory usage while maintaining acceptable detection accuracy.

Existing studies on rice disease detection, such as YOLOv8-based models (Zhan et al., 2024) and lightweight frameworks like LCGSC-YOLO (Wang et al., 2024), primarily focus on accuracy under controlled laboratory conditions. However, these models face significant limitations in real-world agricultural settings. For instance, they often exhibit high computational complexity and large parameter sizes, making deployment on resource-constrained devices impractical. Additionally, models like Faster R-CNN (Ren et al., 2017) and Mask R-CNN (Dorrer and Alekhin, 2021), while accurate, lack real-time capabilities due to their two-stage architecture. Furthermore, existing datasets rarely account for environmental variability such as lighting changes, occlusions, or seasonal variations, leading to poor generalization in field conditions. These limitations underscore the need for a lightweight, adaptive model that balances accuracy with computational efficiency while addressing complex environmental challenges. Disease detection in complex agricultural environments encounters challenges such as high computational resource consumption, stringent real-time requirements, and the need for enhanced detection accuracy. To address these issues, this study chose the latest and relatively stable YOLOv11 model from the YOLO series as the research foundation. The YOLOv11 model has drawn attention for its higher detection accuracy, fewer parameters, and smaller model size. The aim is to further enhance and optimize this model to meet the specific demands of rice disease detection. The proposed YOLOv11-MSDFF-RiceD model, which is the optimized version, holds great potential for integration into large-scale precision agriculture systems. For example, it can be installed on drones with real-time imaging sensors to automatically monitor rice fields, facilitating early disease detection over extensive agricultural areas. Moreover, its lightweight design (only 4.7 MB) enables smooth integration into handheld devices used by farmers for on-site diagnosis. By combining the model with automated pesticide spraying systems, farmers can precisely treat infected areas, reducing chemical usage and operational costs. These applications are in line with the increasing demand for sustainable and intelligent farming practices, providing a scalable solution to minimize crop losses and enhance food security. Through the improvements made to the YOLOv11 model, we expect to develop a rice disease detection model that not only achieves high accuracy but also meets the real-time requirements in detection speed. Considering the limited computing power of mobile devices, we have also placed special emphasis on the lightweight design of the model, aiming to realize efficient disease detection on resource-constrained devices and promote the application of rice disease detection technology in actual agricultural production.

## 2 Materials and methods

### 2.1 Datasets construction

In the field of deep learning, the mobility and generalization ability of the algorithm model are always one of the key challenges. Models showing excellent performance in the laboratory environment often have a significant decrease in recognition efficiency when transferred to the natural environment. In order to solve this problem, this study mainly focuses on the accurate detection of rice leaf diseases and has selected four common rice diseases, including Rice Blast, Brown Spot, Fusarium wilt and Bacterial blight.

**Figure 1:**
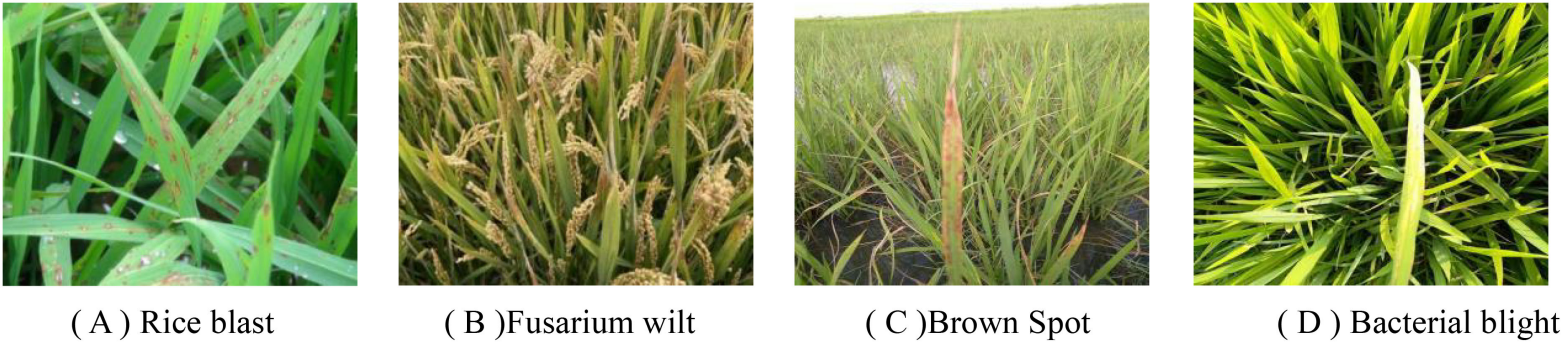
Part of the self-built data set samples. **(A)** Rice blast **(B)** Fusarium wilt **(C)** Brown Spot **(D)** Bacterial blight.

The construction of this data set strictly follows the principles of scientificity and diversity, covering samples widely collected from the Internet and data taken on site to ensure the authenticity and richness of the data set. The data collection was carried out in the high standard farmland demonstration area (32° 44 ′ N, 119° 29 ′ E) in Qinwang Village, Cheluo Town, Gaoyou City, Jiangsu Province from mid-June to late August 2024. In the collection process, we used DJI MAVIC AIR UAV and iPhone 12 smartphone as the main collection tools. In view of the limitation of the endurance of the UAV, we determined the best shooting parameters through multiple flight experiments: the UAV flight speed is 3m/s to 5m/s, the height is 3 to 4 meters from the rice plant, and the mobile phone camera is 30 to 50 cm away from the rice plant. The position is taken to ensure that the collected image is clear and usable. All captured images are saved in JPG format with a resolution of 2720 × 1530 pixels or 1920 × 1080 pixels to ensure a clear presentation of image details. **Figure 1** shows some samples of the data set, and **Table 1** lists the main features of various diseases in detail. In order to enhance the diversity and challenge of the datasets and ensure the model’s robustness, a comprehensive approach was taken during data collection. A variety of natural environments, including soil, sky, paddy fields, as well as complex backgrounds like water reflections and overlapping foliage, were deliberately selected as the background for on - site shooting. The shooting strategies incorporated following light, reversing light, different distances (close and long distance), and multi - angles (pitch angle, elevation angle) to comprehensively simulate various light and perspective conditions. Images were also collected across different seasons, specifically from mid - June to late August, which allowed for the inclusion of seasonal variations. For instance, images of early - stage (yellowing leaves) and late - stage (necrotic lesions) infections were captured. Additionally, different lighting scenarios were considered, such as those at dawn, midday, and dusk, with deliberate inclusion of overcast, sunny, and partially shaded conditions. This extensive coverage of diverse environmental conditions mimics real - world challenges and ensures the model’s adaptability to climatic and environmental heterogeneity, which is a crucial factor for its deployment in precision agriculture systems

**Table 1:** This paper studies rice diseases and their characteristics.

Type	The main characteristics of the disease
Rice blast	Spindle or oval gray-white to brown lesions appeared on the leaves, with yellow halos. Brown dots appeared on stem nodes and panicle necks, which may lead to fracture. Brown oval or irregular spots are formed on the grains (Shang, 2021).
BrownSpot	The lesion usually starts from the leaf tip or leaf margin. At first, it is dark green water stain, and then expands into a short strip spot, and then extends up and down along the leaf margin or midrib to a long strip spot, and finally turns to gray white and curls inward (Feng et al., 2022).
Fusarium wilt	The leaves first appeared dark green, and then the lower leaves expanded from the tip along both sides of the leaf margin to the base to become yellowish brown, and produced many rust-like spots of different sizes of reddish brown or dark brown. Finally, the spots merged into plaques, and the leaves gradually withered (Qi et al., 2021).
Baterial blight	Rice bacterial blight is mainly manifested as yellow-green to dark-green water-soaked stripes on the leaves, and then develops into corrugated spots along the leaf margin or midrib, which can lead to yellowing, curling or wilting of the leaves in severe cases (Qi et al., 2021).

In order to solve the problem of over-fitting or under-fitting of the model caused by the imbalance of the number of images of different disease categories in the data set, and enhance the robustness and generalization ability of the model, this study uses image enhancement technology to expand the data set. The specific enhancement methods include horizontal flipping of the image, random rotation, and random adjustment of brightness and contrast (Zhong et al., 2017). After these enhancement steps and excluding the images with information loss, 13464 disease images were finally obtained. These images are divided into training set, validation set and test set according to the proportion of 70%, 20% and 10%. The number of samples in each part is listed in **Table 2**.

**Table 2:** The number of samples in each part.

Type	Train	Val	Test	Total
Rice blast	2539	648	372	3559
Baterial blight	2918	704	370	3992
Fusarium wilt	951	592	257	1800
BrownSpot	2857	859	397	4113

**Figure 2:**
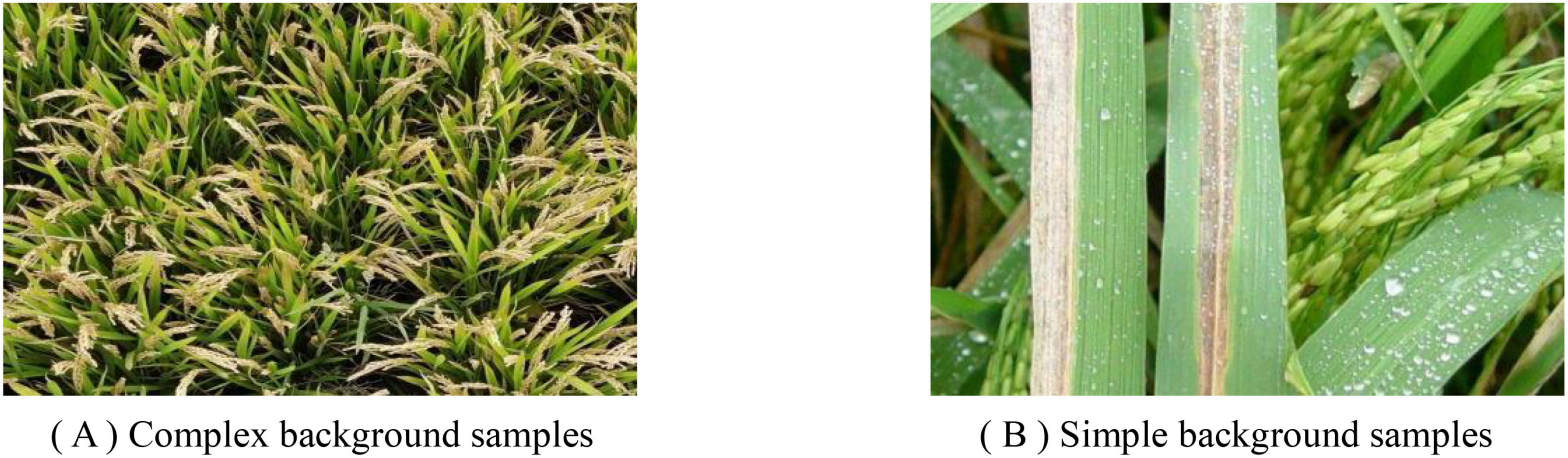
Datasets samples. **(A)** Complex background samples. **(B)** Simple background samples.

In the process of dataset construction, we noticed that the characteristics of bacterial blight often appear as thin strips, which may lead to many non-disease features being incorrectly included in the annotation process, as shown in **Figure 2A**. This mislabeling may cause the model to learn invalid features, which will affect its detection performance. In order to solve this problem, this study decided to introduce more detailed disease images, as shown in **Figure 2B**, to help the model learn more effective features. This method will improve the accuracy of the model ‘s recognition of disease features, thereby improving the overall performance of the model.

### 2.2 YOLOv11 network model

**Figure 3:**
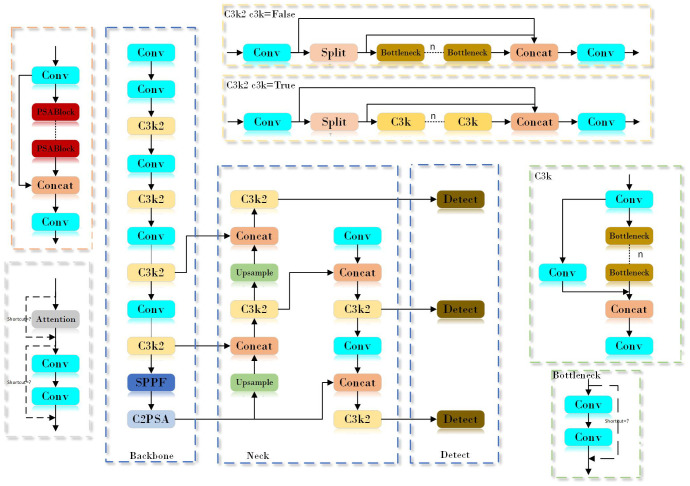
YOLOv11n network structure diagram.

YOLOv11n is a lightweight target detection model in the YOLOv11 series. Compared with the same series of models such as YOLOv11s and YOLOv11x, although there is a compromise in detection accuracy, it has achieved a significant improvement in detection speed. By reducing the amount of calculation and parameters, YOLOv11n reduces the requirements for hardware devices, and effectively improves real-time performance, so that it shows more prominent advantages in scenarios with strict requirements for real-time performance and hardware resources. As shown in **Figure 3**, the network structure of YOLOv11 n is composed of Input, Backbone, Neck and Head. The input end performs image acquisition and preprocessing. By implementing an adaptive scaling strategy, the size of the input image is ensured to match the input requirements of the model. The adaptive anchor frame technology is used to calculate the bounding box that is most suitable for the current image. In addition, the input data is enhanced by using multi-image stitching and cropping techniques to improve the performance and robustness of the model. The backbone network consists of several key modules, including the convolutional layer (Conv), C3k2, SPPF, and C2PSA, which are jointly responsible for extracting feature information from the input image. The C3k2 module is developed on the basis of C2f, which integrates two different parameter configurations: C3k and Bottleneck. The design goal of this module is to improve the accuracy of feature extraction while maintaining computational efficiency and inference speed. The C3k2 module allows switching between C3k and Bottleneck configurations by introducing an optional C3k parameter. When the C3k configuration is enabled, the module enhances the extraction ability of local features by adding two convolution operations, which is particularly useful in complex scenes because it can improve the resolution and expression ability of features. On the contrary, if the C3k parameter is not enabled, the module will adopt the standard Bottleneck configuration, and the function of the C3k2 module is the same as that of C2f. This design flexibility enables the C3k2 module to adjust its structure according to the needs of different tasks. SPPF includes three maximum pooling operations and one convolution operation, which is helpful to realize the effective fusion of global information and local information. C2PSA extends C2f by introducing PSA (Position-Sensitive Attention), aiming to enhance feature extraction ability through multi-head attention mechanism and feedforward neural network. It can selectively add residual structure (shortcut) to optimize gradient propagation and network training effect. The neck network is composed of a path aggregation network (PAN) and a feature pyramid network (FPN), which is mainly used to integrate feature maps from different levels and scales to achieve effective fusion of features. The Head part adopts a decoupling head structure and combines an anchor-free strategy to allow the model to perform image detection and classification tasks independently at different scales.

### 2.3 YOLOv11-MSDFF-RiceD

**Figure 4:**
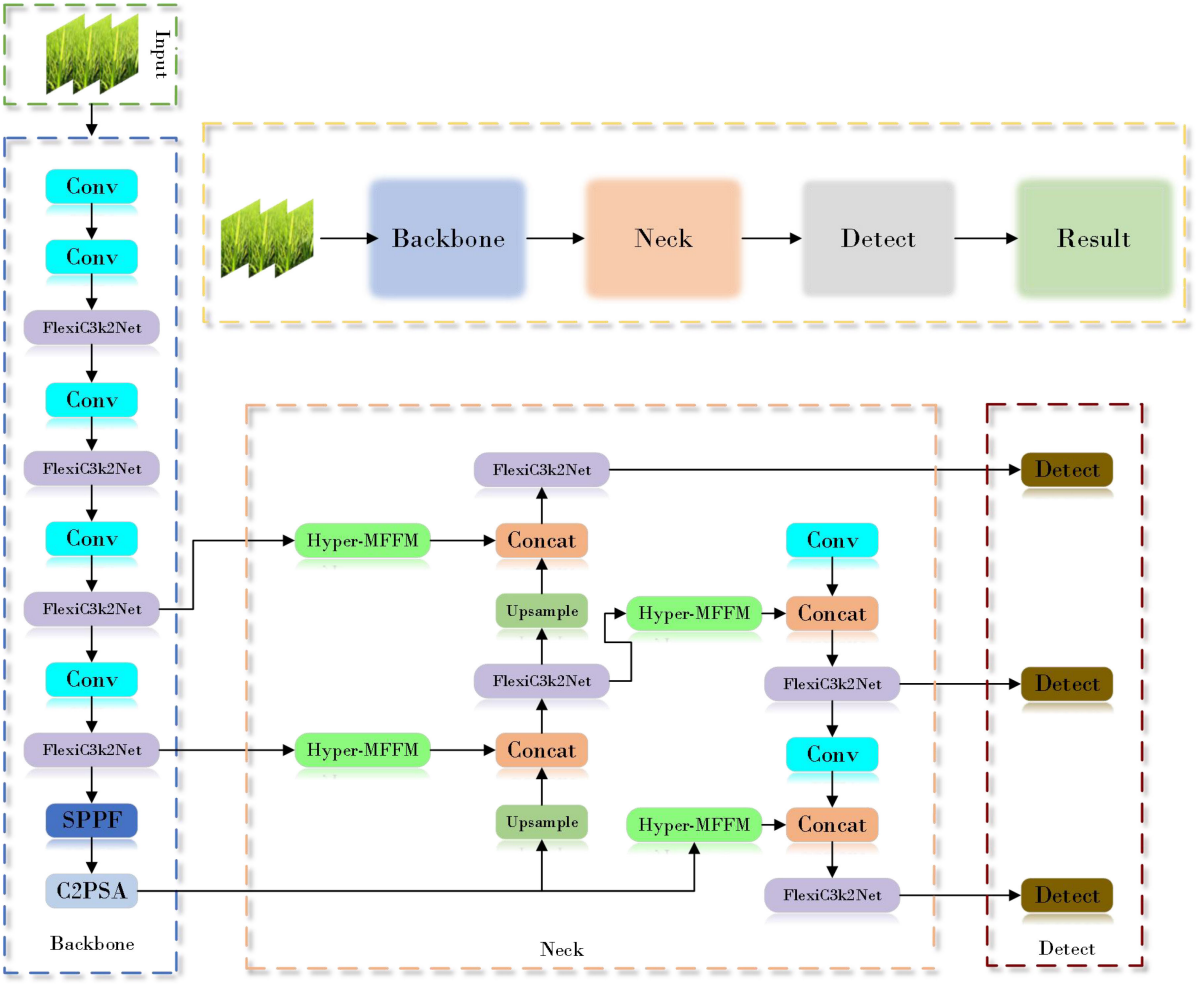
YOLOv11-MSDFF-RiceD network structure diagram.

In order to increase the detection speed and accuracy of the model for rice diseases in complex field environments, this study improved the model based on the original YOLOv11. The network structure is shown in **Figure 4** above.

### 2.4 Optimization of backbone feature extraction

**Figure 5:**
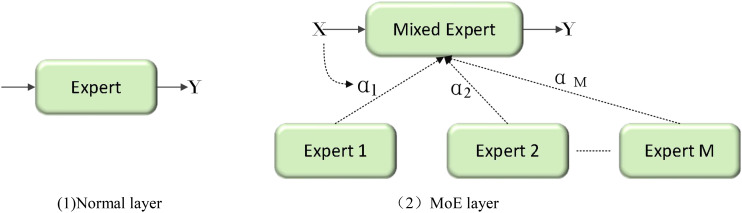
Structure diagram of dynamic convolution. (1) Normal layer. 2) MoE layer.

**Figure 6:**
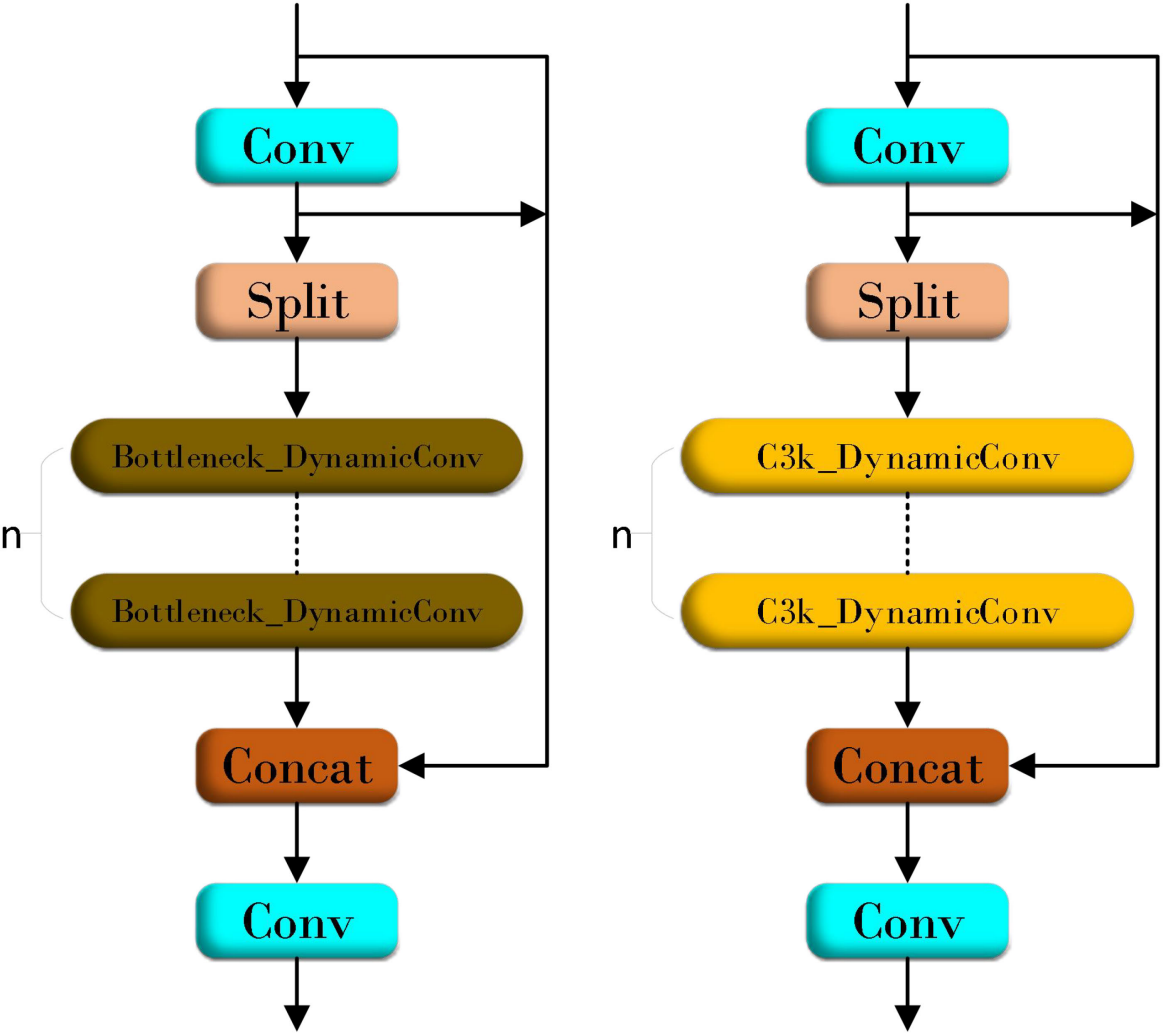
FlexiC3k2Net structure diagram.

As a new lightweight model of low floating-point operations (FLOPs), YOLOv11n has achieved a corresponding improvement in detection speed although it has been damaged in detection accuracy. In order to balance the computational efficiency and detection accuracy of the model in the disease detection task, this study draws on the design idea of ParameterNet (Han et al., 2023). By increasing the number of parameters of large-scale visual pre-training models without significantly increasing FLOPs, the network uses dynamic convolution technology. **Figure 5** shows the structure of dynamic convolution. The dynamic convolution in can significantly enhance the expression ability of the model by using multiple convolution kernels and dynamically adjusting the weight of these convolution kernels according to the input features. This design improves its capacity by integrating multiple dynamic convolution kernels to capture more complex functional relationships. According to its adaptive computer mechanism, the model can automatically adjust the weight of the convolution kernel according to different input features to achieve more flexible and effective feature extraction. Dynamic convolution is used to introduce additional parameters into the network, which only brings a slight increase in FLOPs. This paper uses similar design ideas to innovate the Bottleneck in C3k2 and proposes the FlexiC3k2Net module. **Figure 6** is the FlexiC3k2Net structure diagram.FlexiC3k2Net enhances feature extraction by dynamically adjusting convolutional kernel weights based on input characteristics. Unlike static convolutions, FlexiC3k2Net employs multiple kernels whose contributions are weighted via a lightweight MLP. For example, in detecting thin bacterial blight stripes, the module prioritizes kernels capturing linear patterns, while for larger lesions like rice blast, it emphasizes spatial context. This adaptability reduces redundant computations while improving accuracy for heterogeneous targets.

PNC3k2 has M dynamic convolution kernels, which can be expressed as Equation 1:


(1) $$\text{Y}=\text{X}*{\text{W}}^{\prime },\;\;\;\;\;\;{\text{W}}^{\prime }=\sum_{\text{i}=1}^{\text{M}}{\text{α}}_{\text{i}}{\text{W}}_{\text{i}}$$

Among them, $W_{i}\in R^{C_{out}\times C_{in}\times H\times W}$ represents the weight tensor of the i th convolution kernel, and $\alpha_{i}$ is the dynamic coefficient corresponding to the convolution kernel. These coefficients $\alpha_{i}$ are calculated dynamically through a multi-layer perceptron (MLP) module based on the different characteristics of the input samples and are expressed as Equation 2:


(2) α=softmax(MLP(Pool(X))),

In comparison with the original convolutional layer, the coefficient generation in Formula 2 only leads to a slight increase in the number of floating-point operations (FLOPS). Therefore, the PNC3k2 implemented by dynamic convolution can significantly reduce the growth of FLOPs while introducing a large number of additional parameters.

In the ordinary convolution layer, the total number of parameters is ${\text{C}}_{\text{out}}\cdot {\text{C}}_{\text{in}}\cdot \text{K}\cdot \text{K}$ , and the corresponding floating-point operations (FLOPs) are ${\text{H}}^{\prime }\cdot {\text{W}}^{\prime }\cdot {\text{C}}_{\text{out}}\cdot {\text{C}}_{\text{in}}\cdot \text{K}\cdot \text{K}$ . In contrast, the dynamic convolution architecture enhances the parameter efficiency and computational performance of the model by integrating the coefficient generation module, the dynamic weight fusion mechanism and the convolution execution process. Specifically, the coefficient generation module is conFigd with ${\text{ C}}_{\text{in}}$ hidden units, which requires ${\text{C}}_{\text{in}}^{2}+{\text{C}}_{\text{in}}\text{M}$ parameters and consumes ${\text{C}}_{\text{in}}^{2}+{\text{C}}_{\text{in}}\text{M}$ FLOPs to dynamically derive the coefficients of the convolution kernel. Although the dynamic weight fusion process does not increase the parameter burden of the model, it involves $\text{M}\cdot {\text{C}}_{\text{out}}\cdot {\text{C}}_{\text{in}}\cdot \text{K}\cdot \text{K}$ FLOPs to achieve real-time combination of weights. Combining these components, the total number of parameters of the dynamic convolutional layer and the amount of FLOPs calculation are increased to ${\text{C}}_{\text{in}}^{2}+{\text{C}}_{\text{in}}\text{M}+\text{M}\cdot {\text{C}}_{\text{out}}\cdot {\text{C}}_{\text{in}}\cdot \text{K}\cdot \text{K}$ and ${\text{C}}_{\text{in}}^{2}+{\text{C}}_{\text{in}}\text{M}+\text{M}\cdot {\text{C}}_{\text{out}}\cdot {\text{C}}_{\text{in}}\cdot \text{K}\cdot \text{K}+{\text{H}}^{\prime }\cdot {\text{W}}^{\prime }\cdot {\text{C}}_{\text{out}}\cdot {\text{C}}_{\text{in}}\cdot \text{K}\cdot \text{K}$ , respectively. This design not only improves the adaptability of the model to the input data, but also achieves the goal of increasing the complexity of the model while maintaining the computational efficiency through refined parameter management and computational optimization.

The parameter ratio of dynamic convolution to standard convolution is (Equation 3):


(3) $${\text{R}}_{\text{p}\text{a}\text{r}\text{a}\text{m}}=\frac{{\text{C}}_{\text{i}\text{n}}^{2}+{\text{C}}_{\text{i}\text{n}}\text{M}+\text{M}{\text{C}}_{\text{o}\text{u}\text{t}}{\text{C}}_{\text{i}\text{n}}{\text{K}}^{2}}{{\text{C}}_{\text{o}\text{u}\text{t}}\cdot {\text{C}}_{\text{i}\text{n}}\cdot \text{K}\cdot \text{K}}=\frac{{\text{C}}_{\text{i}\text{n}}}{{\text{C}}_{\text{o}\text{u}\text{t}}{\text{K}}^{2}}+\frac{\text{M}}{{\text{C}}_{\text{o}\text{u}\text{t}}{\text{K}}^{2}}+\text{M}\approx \frac{1}{{\text{K}}^{2}}+\text{M}.\left(\text{M}\ll {\text{C}}_{\text{o}\text{u}\text{t}}{\text{K}}^{2},\;{\text{C}}_{\text{i}\text{n}}\approx {\text{C}}_{\text{o}\text{u}\text{t}}\right)$$

The proportion of FLOPs is (Equation 4):


(4) $$\begin{array}{l} {\text{R}}_{\text{f}\text{l}\text{o}\text{p}\text{s}}=\frac{{\text{C}}_{\text{i}\text{n}}^{2}+{\text{C}}_{\text{i}n}\text{M}+\text{M}{\text{C}}_{\text{o}\text{u}\text{t}}{\text{C}}_{\text{i}\text{n}}{\text{K}}^{2}+{\text{H}}^{\prime }\cdot {\text{W}}^{\prime }\cdot {\text{C}}_{\text{o}\text{u}\text{t}}\cdot {\text{C}}_{\text{i}\text{n}}\cdot \text{K}\cdot \text{K}}{{\text{H}}^{\prime }\cdot {\text{W}}^{\prime }\cdot {\text{C}}_{\text{o}\text{u}\text{t}}\cdot {\text{C}}_{\text{i}\text{n}}\cdot \text{K}\cdot \text{K}}=\frac{{\text{C}}_{\text{i}\text{n}}}{{\text{H}}^{\prime }\cdot {\text{W}}^{\prime }\cdot {\text{C}}_{\text{o}\text{u}\text{t}}\cdot \text{K}\cdot \text{K}}+\frac{\text{M}}{{\text{H}}^{\prime }\cdot {\text{W}}^{\prime }\cdot {\text{C}}_{\text{o}\text{u}\text{t}}\cdot \text{K}\cdot \text{K}}+\\ \frac{\text{M}}{{\text{H}}^{\prime }\cdot {\text{W}}^{\prime }}+1\approx 1,(1<\text{M}\ll {\text{H}}^{\prime }\cdot {\text{W}}^{\prime },\;\;\;\;{\text{C}}_{\text{o}\text{u}\text{t}}\approx {\text{C}}_{\text{i}\text{n}}) \end{array}$$

Therefore, compared with standard convolution, dynamic convolution has about M times the parameters, and the additional FLOPs can be ignored.

### 2.5 Efficient multi-scale feature fusion module

**Figure 7:**
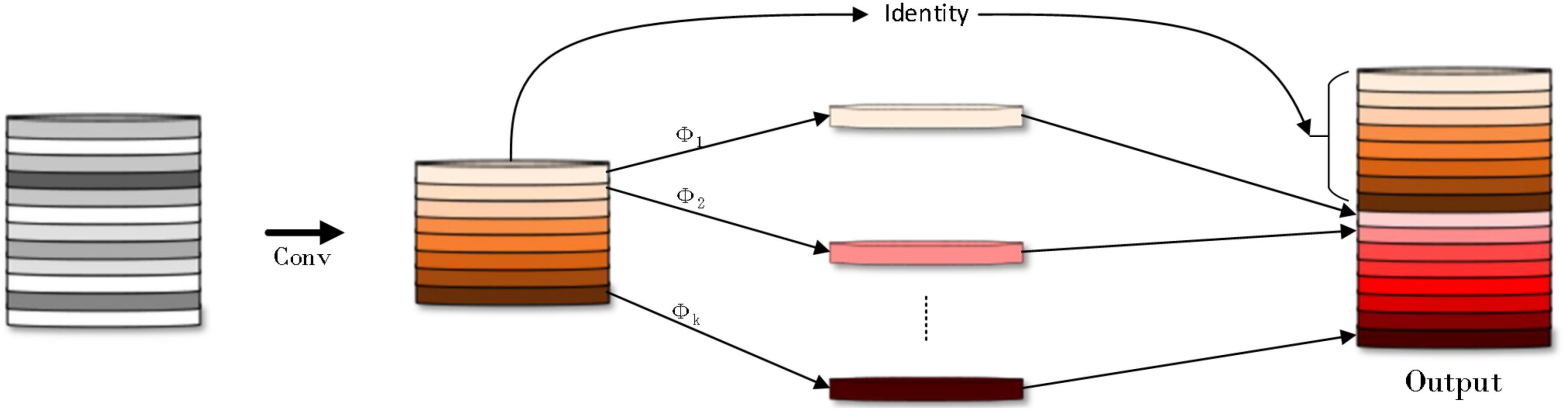
GhostNet structure diagram.

**Figure 8:**
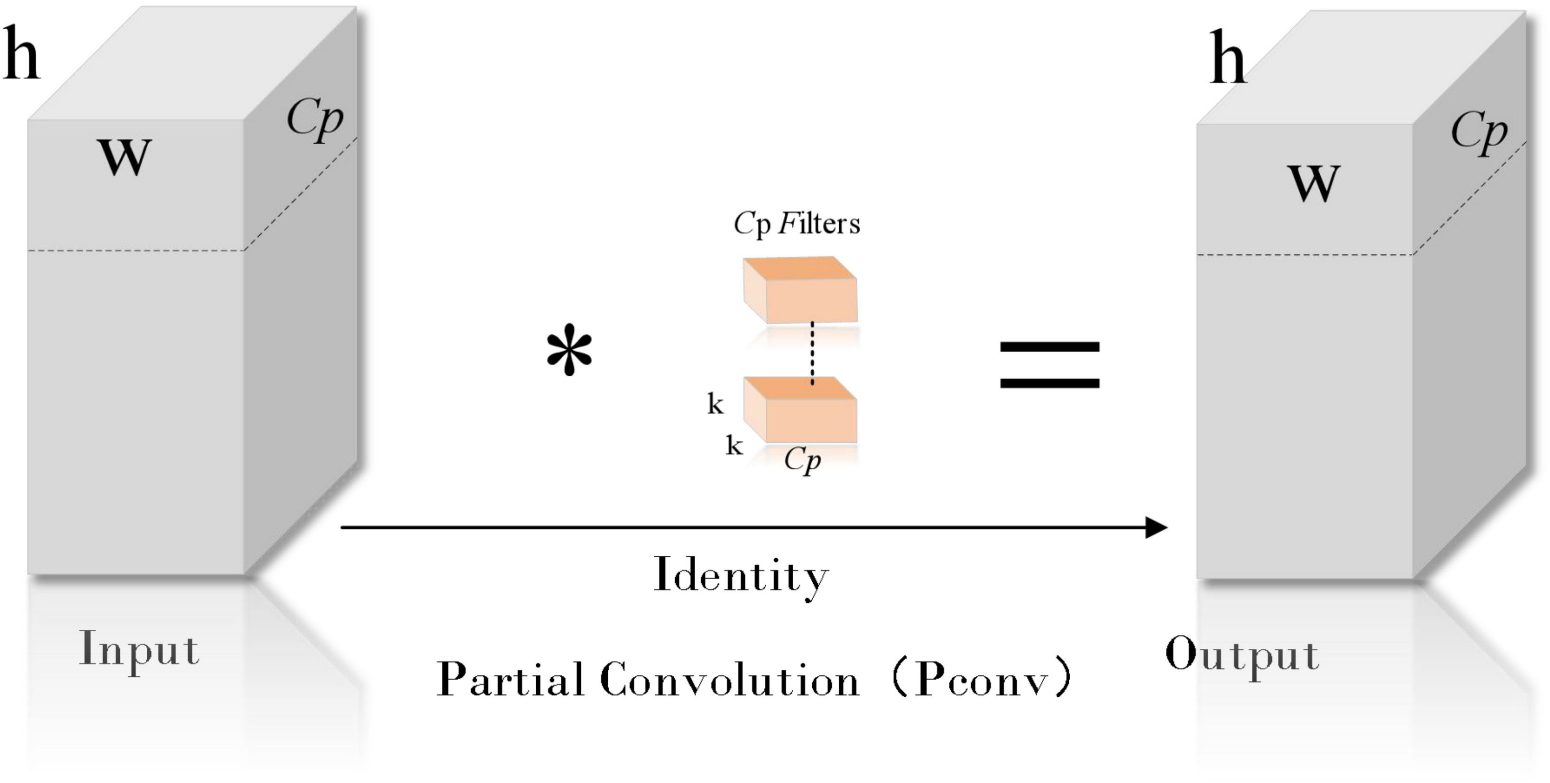
Pconv structure diagram.

In the rice disease detection task, the coexistence of small target lesions and large targets (such as healthy leaves) poses a challenge to model training. In the training process, the model may tend to focus on the big target and ignore the small target lesions, resulting in insufficient capture of the contextual features of the small target. This bias may reduce the recognition accuracy of the model for small target lesions and increase the risk of missed or false detection. At the same time, the existence of large targets also introduces a large amount of redundant information, which increases the learning burden of the model. In order to solve this problem, this study proposes an efficient multi-scale feature fusion module (Efficient multi-scale feature fusion module, EMFFM). The design of the module draws on the design concepts of GhostNet (Han et al., 2020) (**Figure 7**) and FasterNet (Chen et al., 2023) (**Figure 8** where * represents the meaning of multiplication).

The core idea of GhostNet is to decompose the traditional convolutional layer into two smaller convolutional layers: one is the ghost convolutional layer, which only uses a part of the original convolutional layer for calculation; the other is the residual convolution layer, which is responsible for processing the output of the remaining channels.

FasterNet introduces the concept of Partial Convolution to extract spatial features more efficiently by reducing redundant computation and memory access.

**Figure 9:**
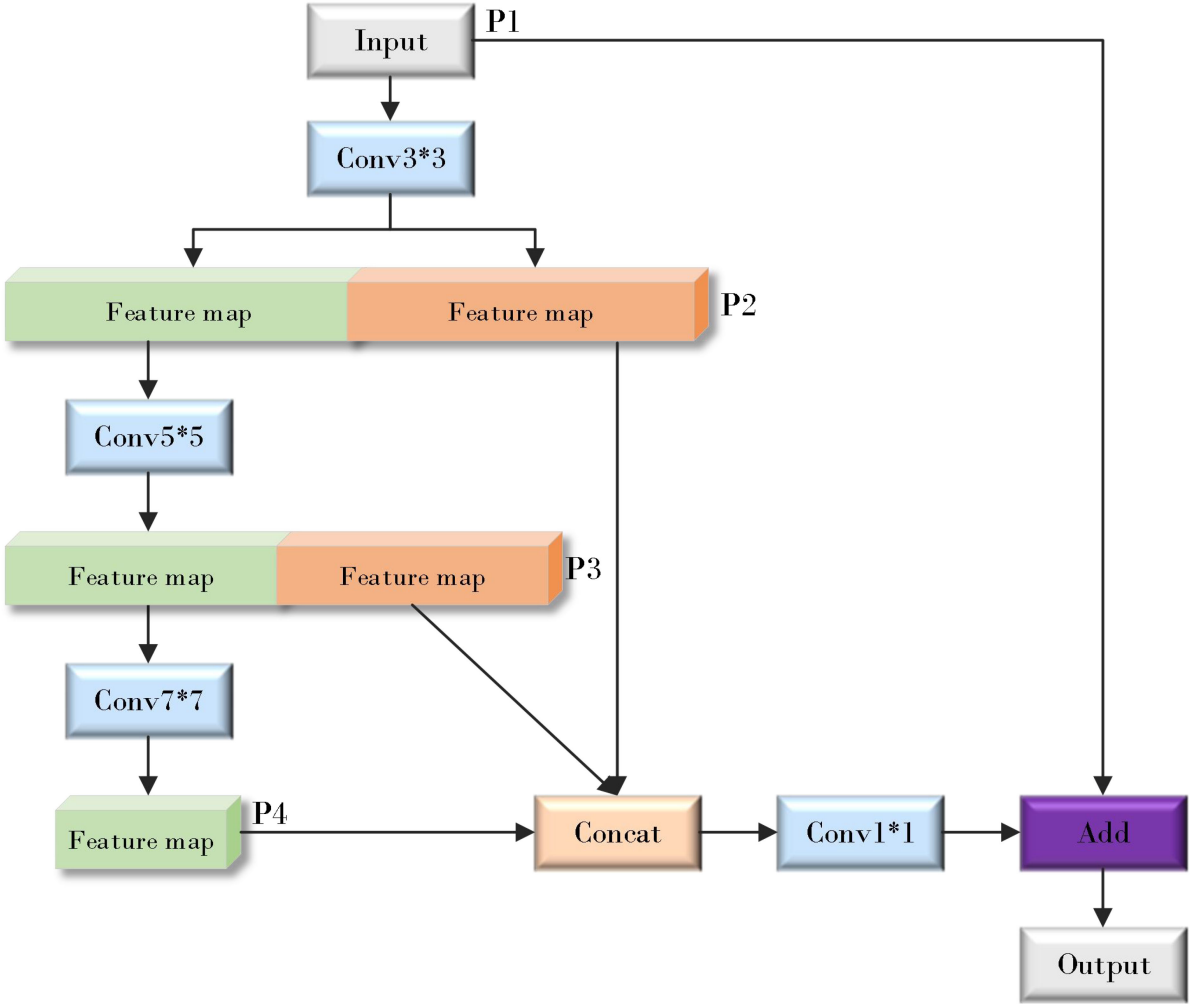
Efficient multi-scale feature fusion module (EMFFM).

The design of EMFFM combines these two network design concepts. As shown in **Figure 9** (where * represents the meaning of multiplication) below, the input image is first processed by a 3x3 convolutional layer and then divided into two sets of features: one set of features continues to be processed by a 5x5 convolutional layer, while the other set of features is retained for subsequent feature fusion. After multiple convolution operations, the feature information will inevitably be lost, so the features of P2, P3 and P4 layers are partially fused. However, this operation is only carried out on some channels, which improves the computational efficiency. Finally, the features of different scales are fused by 1x1 convolution layer, and the input features are added to the processed features by residual connection, which effectively retains the original information and introduces new multi-scale information, and enhances the expression ability of the model.

### 2.6 Loss function

In YOLOv11, CIoU (distributed focusing loss function) is used as the regression loss function of the detection box, and the matching accuracy is improved by considering the overlapping area, center distance and aspect ratio between the target boxes. Compared with the traditional IoU loss function, the computational complexity of the CIoU loss function is higher, because it requires additional calculation of the distance and angle differences between the target detection boxes, which will increase the calculation time and resource consumption. And CIoU may have limitations when dealing with small targets. Due to the small size of small targets, the difference of bounding box distance and angle between them is relatively small, which makes it difficult for the CIoU loss function to effectively distinguish the subtle differences between these small targets.

**Figure 10:**
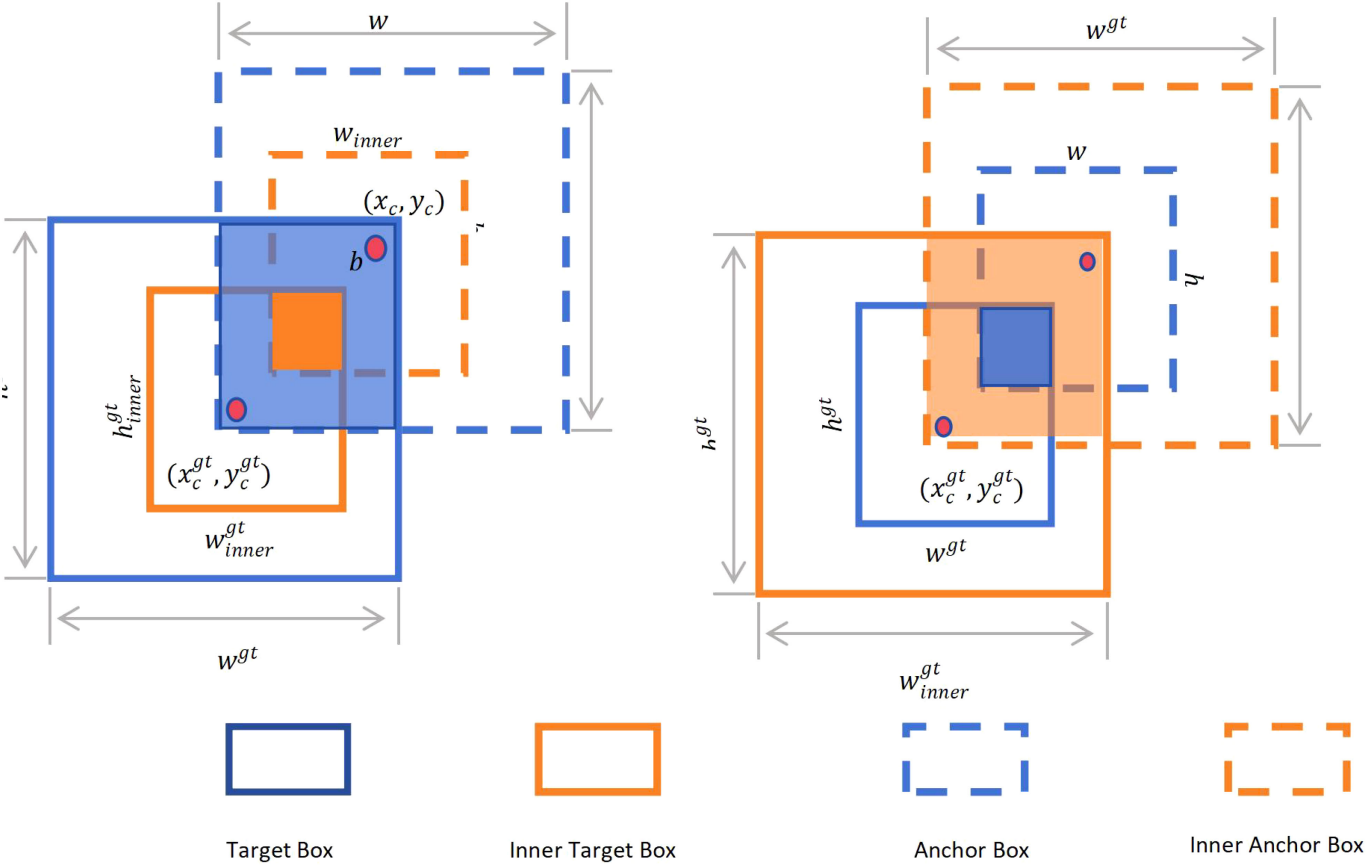
Inner-IoU diagram.

In this study, we refer to the concept of Inner-IoU (Zhang et al., 2023). By introducing multi-scale auxiliary bounding boxes, the concept allows these bounding boxes to be dynamically adjusted according to the sample characteristics to improve the efficiency of bounding box regression. At the same time, the scale factor ratio parameter is added, which can adjust the size of the auxiliary bounding box, and can be optimized for different data sets and detectors, thereby improving the computational performance of the loss function. Inspired by these ideas, we designed Inner-WIoUv2. **Figure 10** below is a diagram of Inner-IoU. As shown in the Figure, the Ground Truth (GT) and Anchor are represented as ${\text{B}}^{\text{gt}}\;$ and B , respectively. The center point inside the GT bounding box and its corresponding GT bounding box itself are represented by $\left({\text{x}}_{c}^{\text{gt}},{\text{y}}_{c}^{\text{gt}}\right)$ . The center point inside the anchor box and its corresponding anchor box are represented by $\left({\text{x}}_{\text{c}},{\text{y}}_{\text{c}}\right)$ . The width and height of the GT bounding box are represented by ${\text{ w}}^{\text{gt}}$ and ${\text{ h}}^{\text{gt}}$ , respectively, while the width and height of the anchor box are represented by w and h , respectively. The scale factor, usually expressed as ‘ratio’, ranges from [0.5, 1.5].

In addition, the definition of Inner-loU is as follows (Equations 5–11):


(5) $${\text{b}}_{\text{l}}^{\text{ɡ}\text{t}}={\text{x}}_{\text{c}}^{\text{ɡ}\text{t}}-\frac{{\text{w}}^{\text{ɡ}\text{t}}*\text{r}\text{a}\text{t}\text{i}\text{o}}{2},{\text{b}}_{\text{r}}^{\text{ɡ}\text{t}}={\text{x}}_{\text{c}}^{\text{ɡ}\text{t}}+\frac{{\text{w}}^{\text{ɡ}\text{t}}*\text{ratio}}{2}$$

(6) $${\text{b}}_{\text{t}}^{\text{ɡ}\text{t}}={\text{y}}_{\text{c}}^{\text{ɡ}\text{t}}-\frac{{\text{h}}^{\text{ɡ}\text{t}}*\text{ratio}}{2},{\text{b}}_{\text{b}}^{\text{ɡ}\text{t}}={\text{x}}_{\text{c}}^{\text{ɡ}\text{t}}+\frac{{\text{w}}^{\text{ɡ}\text{t}}*\text{ratio}}{2}$$

(7) $${\text{b}}_{\text{l}}={\text{x}}_{\text{c}}-\frac{\text{w}*\text{ratio}}{2},{\text{b}}_{\text{r}}={\text{x}}_{\text{c}}+\frac{\text{w}*\text{ratio}}{2}$$

(8) $${\text{b}}_{\text{t}}={\text{y}}_{\text{c}}-\frac{\text{h}*\text{ratio}}{2},{\text{b}}_{\text{b}}={\text{y}}_{\text{c}}+\frac{\text{h}*\text{ratio}}{2}$$

(9) $$\text{inter}=\left(\text{min}\left({\text{b}}_{\text{r}}^{\text{ɡ}\text{t}},{\text{b}}_{\text{r}}\right)-\text{max}\left({\text{b}}_{\text{l}}^{\text{ɡ}\text{t}},{\text{b}}_{\text{l}}\right)\right)*\left(\text{min}\left({\text{b}}_{\text{b}}^{\text{ɡ}\text{t}},{\text{b}}_{\text{b}}\right)-\text{max}\left({\text{b}}_{\text{t}}^{\text{ɡ}\text{t}},{\text{b}}_{\text{t}}\right)\right)$$

(10) $$\text{union}=\left({\text{w}}^{\text{ɡ}\text{t}}*{\text{h}}^{\text{ɡ}\text{t}}\right)*{\left(\text{ratio}\right)}^{2}+\left(\text{w}*\text{h}\right)*{\left(\text{ratio}\right)}^{2}-\text{inter}$$

(11) $$\text{Io}{\text{U}}^{\text{inner}}=\frac{\text{inter}}{\text{union}}$$

WIoUv2 (Tong et al., 2023) The bounding box regression loss function is constructed to reduce the loss effect on simple samples, and a monotonic focusing coefficient is introduced so that the model can process difficult samples more intensively, thereby improving the target detection performance. The formula of the loss function is shown in Equation 12, which aims to optimize the training effect of the model and highlights the superiority in the face of challenging target detection tasks.


(12) $${\text{ℒ}}_{\text{WIoUv}2}={\text{ℒ}}_{\text{I}\text{o}\text{U}}^{{\text{r}}^{*}}{\text{ℒ}}_{\text{WIoUv}1},\;\text{r}>0$$

In the process of model training, ${ℒ}_{\text{IoU}}^{{\text{r}}^{*}}$ in the above formula may decrease the convergence speed with the gradual decrease of loss function ${ℒ}_{\text{IoU}}$ , which may lead to the slow convergence of the model in the later training stage. In order to deal with this challenge, we introduce the moving average $\bar{{ℒ}_{\text{IoU}}}$ , which can effectively maintain the overall loss function at a relatively high level, thus promoting the stable training and faster convergence of the model. As shown in Equation 13:


(13) $${\text{ℒ}}_{\text{W}\text{I}\text{o}\text{U}\text{v}2}={\left(\frac{{\text{ℒ}}_{\text{IoU}}^{{\text{r}}^{*}}}{{\text{ℒ}}_{\text{IoU}}}\right)}^{\text{r}}{\text{ℒ}}_{\text{WIoUv}1}$$

According to the above formula, the calculation formula of Inner-WIoU is (Equation 14):


(14) $${\text{ℒ}}_{\text{Inner}-\text{WIoUv}2}={\text{ℒ}}_{\text{WIoUv}2}+\text{IoU}-\text{Io}{\text{U}}^{\text{Inner}}$$

**Figure 11:**
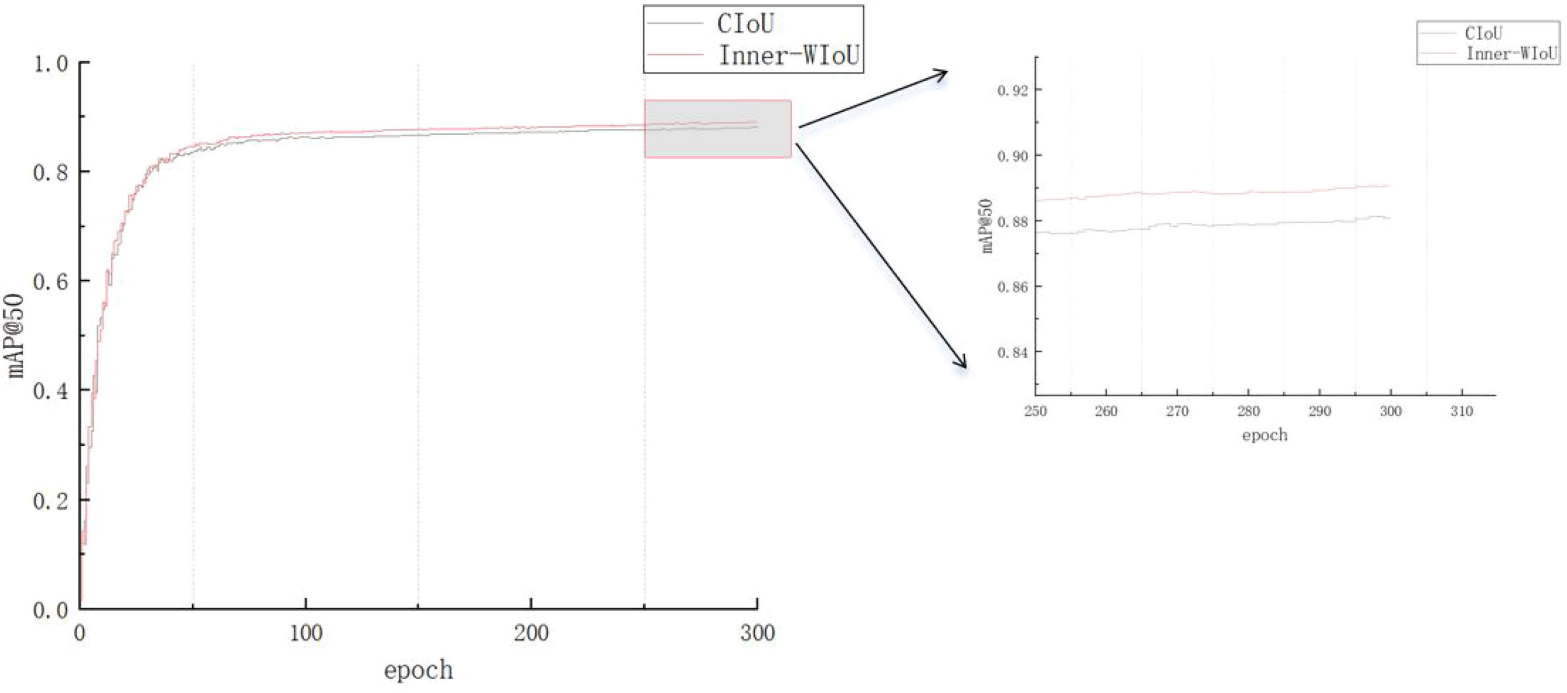
Comparison of experimental results of different loss functions.

It can be seen from **Figure 11** that after the network is added such as Inner-WIoUv2, the accuracy is significantly improved.

### 2.7 Model pruning

**Figure 12:**
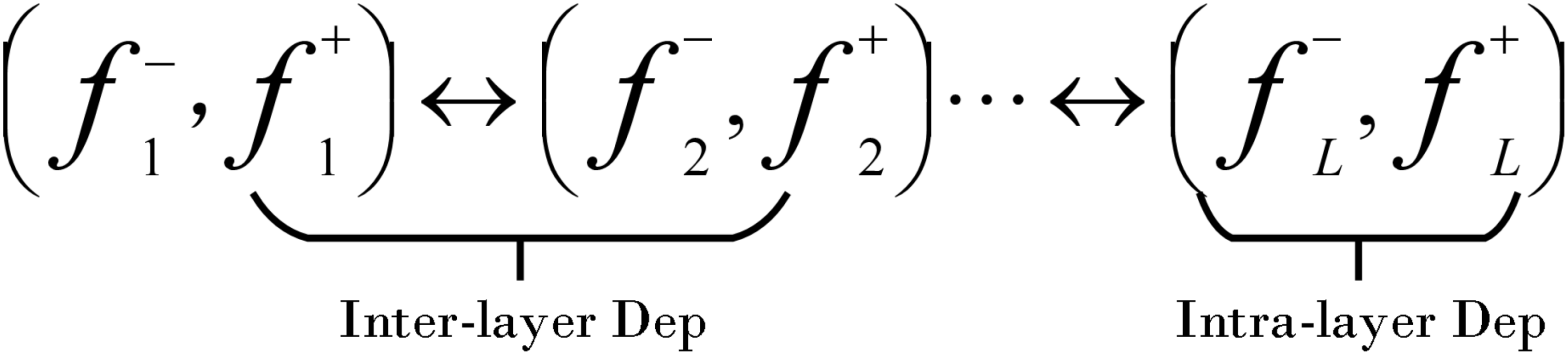
CNN in DepGraph.

In order to optimize the neural network structure and reduce the computational resource consumption on resource-constrained embedded devices, this paper adopts a model pruning method based on Dependency Graph [DepGraph (Fang, 2023)]. This method first reconstructs the convolutional neural network (CNN) into a graph structure, as shown in **Figure 12**. In this structure, we can identify two key dependencies: one is the inter-layer dependency between layers, and the other is the intra-layer dependency within a single layer. Through this graph structure, the network can be decomposed into smaller and more basic components, which helps us to understand and model these dependencies more accurately.

**Figure 13:**
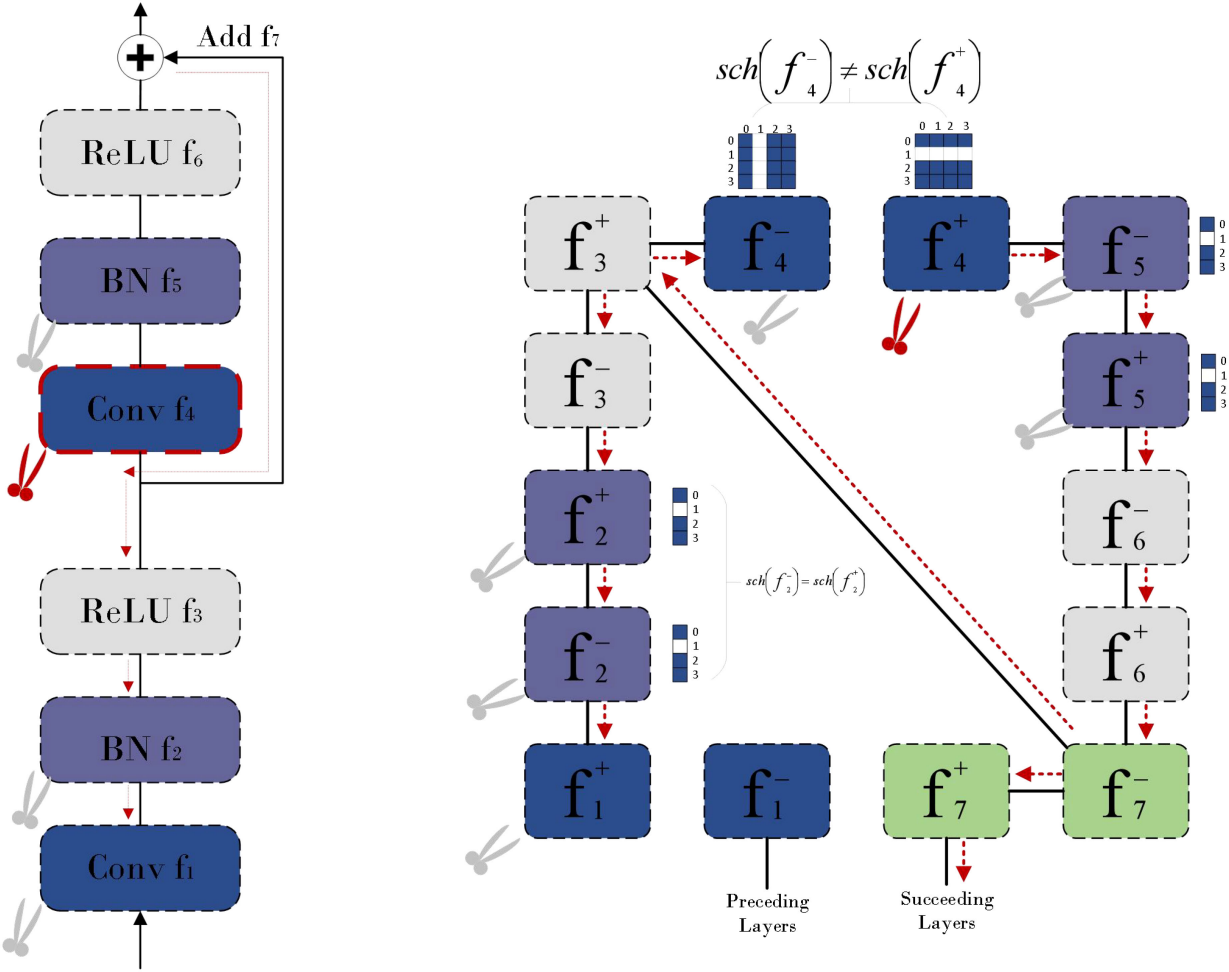
The pruning method of DepGraph.

Then, based on this decomposition, a dependency graph is constructed, which records the direct dependencies between adjacent layers as a simplified representation of network dependencies. Finally, DepGraph (**Figure 13**) groups the layers with dependencies according to the dependency graph, and performs pruning operations at the group level to ensure that if the parameters in a group are pruned, all the parameters of the entire group will be pruned, thereby maintaining the integrity of the network structure and achieving effective structural pruning. Through this method, we can effectively reduce the amount of calculation and parameters of the model while maintaining the expression ability of the model, making it more suitable for deployment on edge computing devices.

## 3 Experimental environment and evaluation index

### 3.1 Experimental environment and parameter settings

The hardware equipment of this research experiment is based on Windows system, RTX4090 graphics card, 24 G graphics memory, Intel i7–13700 K CPU. The deep learning development environment is Pytorch2.2.0 + CUDA11.8 + Python3.10. The deep learning software used is publicly available and can be found on GitHub or other open-source platforms. After many experiments, the most suitable training hyperparameters for this study were found. The specific parameter settings are shown in **Table 3**.

**Table 3:** Deep learning hyperparameters.

Parameter	Value
Image size	640
Batch size	32
learning rate	0.01
epoch	300

### 3.2 Evaluation indicators

In this paper, the performance of the model is evaluated using key indicators such as mean Average Precision (mAP), computational complexity, parameter size, and model size. Among them, the mean average precision (mAP) is used as the core evaluation index to quantify the accuracy performance of the model in multi-category target detection tasks. Specifically, the calculation of recall, precision and average precision is based on the statistical data of True Positives (TP), False Positives (FP) and False Negatives (FN). The determination of mAP is achieved by drawing the Precision-Recall Curve (P-R Curve) and calculating the area under the curve, and then summarizing the average of all categories. Through the comprehensive consideration of these indicators, the performance of the model can be comprehensively evaluated and its performance in different application scenarios can be deeply understood. The calculation formulas of accuracy rate P, recall rate R and average accuracy mAP are as follows (Equations 15–17):


(15) $$\text{P}=\frac{\text{TP}}{\text{TP}+\text{FP}}$$

(16) $$\text{R}=\frac{\text{TP}}{\text{TP}+\text{FP}}$$

(17) $$\text{mAP}=\frac{1}{\text{n}}\sum_{\text{i}=1}^{\text{N}}\text{AP}\left(\text{i}\right)$$

### 3.3 Ablation experiment

In order to verify the advantages of the improved method proposed in this study in the field of rice disease detection, this study designed ablation experiments to evaluate the contribution of each improved module. The experiment includes a total of 8 verification schemes, and all experiments are carried out under a unified hardware environment and experimental parameters. The experimental results are detailed in **Table 4**. The first four groups of experiments introduced FlexiC3k2Net module, EMFFM module and Inner-WIoU module respectively. The results showed that the addition of these modules increased the mAP @ 0.5 index by 0.6%, 0.4% and 1% respectively. In the subsequent experiments, these improved modules are gradually combined and integrated into the model. Finally, compared with the original YOLOv11 model, although the improved YOLOv11-MSDFF-RiceD model has increased in the number of parameters, it has achieved 2.3% and 2.2% improvement in the two key performance indicators of mAP @ 0.5 and mAP @ 0.5: 0.9, respectively. The experimental results show that the proposed improved method has significant performance advantages in rice disease detection tasks.

**Table 4:** Data comparison of ablation experiments.

Treatment	mAP@0.5/%	mAP@0.5:0.9/%	Parameter/M	GFLOPs
YOLOv11	88.1	68.4	2.58	6.3
YOLOv11+FlexiC3k2Net	88.7	70.1	3.46	6.3
YOLOv11+EMFFM	88.5	69.8	2.63	5.8
YOLOv11+Inner-WIoU	89.1	70.6	2.59	6.4
YOLOv11+FlexiC3k2Net+EMFFM	89.4	70.3	3.64	5.9
YOLOv11+FlexiC3k2Net+Inner-WIoU	89.7	70.1	3.67	6.4
YOLOv11+Inner-WIoU+EMFFM	89.5	70	2.71	6.0
YOLOv11-MSDFF-RiceD	90.4	70.6	3.46	6.1

### 3.4 Comparative experiments of different loss functions

In order to verify that the loss function proposed in this paper has certain advantages for disease detection tasks, we systematically compared and analyzed the performance of six different loss functions (CIoU, DIoU (Zheng et al., 2019), EIoU (Zhang et al., 2021), GIoU (Rezatofighi et al., 2019), SIoU (Gevorgyan, 2022), Inner-WIoU) in rice disease detection tasks. The detailed experimental results are shown in **Table 5**.

**Table 5:** Comparative experimental data of different loss functions.

Loss function	Precision/%	Recall/%	mAP@0.5/%	mAP@0.5:0.9/%
CIoU	94.5	82.6	88.1	68.4
DIoU	94.5	83.4	88.5	68.8
EIoU	91.7	82.7	87.8	64.9
GIoU	93.6	83.5	88.7	68.7
SIoU	92.5	84.5	88.7	68.6
Inner-WIoU	94.1	84.8	89.1	70.6

The performance of these loss functions is evaluated by Precision, Recall, and average precision at two different thresholds (mAP @ 0.5 and mAP @ 0.5: 0.9). The results show that CIoU and DIoU are the closest in accuracy, 94.5% and 94.5% respectively, but DIoU is higher in recall rate, 83.4%, while CIoU is 82.6%. EIoU is slightly lower in accuracy, 91.7%, and performs worst on mAP @ 0.5: 0.9, only 64.9%. GIoU and SIoU are relatively close in all indicators, but SIoU is slightly lower at mAP @ 0.5: 0.9, which is 68.6%, while GIoU is 68.7%. Inner-WIoU is not as good as CIoU and DIoU in accuracy, which is 94.1%, but it exceeds other loss functions in recall rate, mAP @ 0.5, mAP @ 0.5: 0.9. The experimental results show that the Inner-WIoU loss function is helpful to improve the efficiency of rice disease detection.

### 3.5 Pruning experiment

When studying the effect of different compression ratios on the performance of the disease detection model, we conducted six experiments with different compression ratios. The experimental results are shown in **Table 6**. The experimental data show that with the increase of compression ratio, the parameters, computing requirements and storage space of the model are reduced, but the performance of the model is also reduced. When the compression ratio is 2, the parameters, computation and storage space of the model are reduced by 25.4%, 49.1% and 36.9% respectively compared with the original model, while the accuracy, mAP @ 0.5 and mAP @ 0.5: 0.9 are only reduced by 1.4%, 0.6% and 0.5% respectively. Therefore, while significantly reducing hardware requirements, the loss of model accuracy is small. We use a pruning method with a compression ratio of 2 to optimize the model.

**Table 6:** Effects of different compression ratios on model performance.

Compression	Precision	Recall	mAP@0.5	mAP@0.5	Parameters	GFLOPs	Model size
ratio	/%	/%	/%	:0.9/%	/M	/G	/MB
/	95.1	84.8	90.4	70.6	3.46	6.1	7.45
2	93.9	84.8	89.8	70.1	2.58	3.1	4.7
2.5	92.5	83.1	88.7	68.8	2.1	2.7	3.5
3	90.8	82.4	88	65.4	1.7	2.6	2.9
3.5	87.3	79	86.2	62.8	1.2	2.3	2.3
4	85.2	76.4	85.6	60.1	0.9	1.8	1.9

### 3.6 Comparative experiments of different models

In order to further evaluate the performance difference between YOLOv11-MSDFF-RiceD and the current mainstream target detection algorithms, this paper selects key indicators such as the number of parameters, the amount of calculation, mAP @ 0.5, mAP @ 0.5: 0.9, accuracy, recall rate and model size, and compares YOLOv11-MSDFF-RiceD with YOLOv5n, YOLOv6n, YOLOv8n, YOLOv9t, YOLOv10n and YOLOv11n on the self-defined data set. The experimental results are summarized in **Table 7**. The results showed that the mAP @ 0.5 of YOLOv11-MSDFF-RiceD reached 89.8%, which was 1.7 percentage points higher than that of YOLOv11n, and 2%, 3.4%, 1.8%, 1.1% and 1.9% higher than that of YOLOv5n, YOLOv6n (Li et al., 2022), YOLOv8n, YOLOv9t (Wang et al., 2024c) and YOLOv10n (Wang et al., 2024a), respectively. This shows that YOLOv11-MSDFF-RiceD performs best in average accuracy, showing its excellent ability in disease detection. In addition, the model size and parameter number of YOLOv11-MSDFF-RiceD were reduced to 4.7 MB and 1.3 million, respectively, which was 36.9% and 49.6% lower than that of YOLOv11n, and showed significant optimization effect in comparison with other detection models.

**Table 7:** Comparative experiments of different models.

Modules	Parameters	GFLOPs	mAP@0.5	mAP@0.5:0.9	Precision	Recall	Size
YOLOv5n	2.18M	5.8G	87.8%	65.5%	91.8%	82.6%	12.7MB
YOLOv6n	4.16M	11.6G	86.4%	68.4%	92.7%	83.5%	8.18MB
YOLOv8n	2.68M	6.8G	88%	68.3%	92.5%	84.3%	7.98MB
YOLOv9t	6.19M	22.1G	88.7%	69%	92.7%	**85.1**%	6.01MB
YOLOv10n	2.26M	6.5G	87.9%	59.6%	89.4%	79.1%	7.16MB
YOLOv11n	2.58M	6.3G	88.1%	68.4%	**94.5**%	82.6%	7.45MB
YOLOv11-MSDFF-RiceD	**1.3M**	**3.1G**	**89.8**%	**70.1**%	93.9%	84.8%	**4.7MB**

The optimal data for each term is expressed in bold.

**Figure 14:**
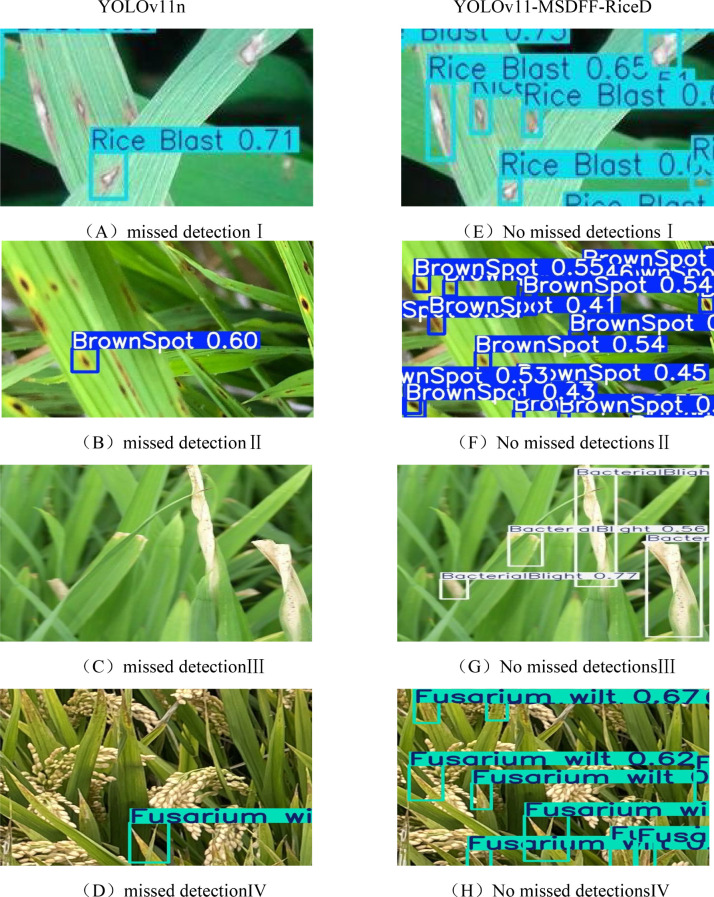
**(A-H)** Details of detection effect.

In this study, we conducted a detailed comparison of the detection performance between YOLOv11-MSDFF-RiceD and YOLOv11n to better understand their capabilities in complex farmland environments. To achieve a more accurate evaluation, we enlarged and cropped images for closer inspection, as shown in **Figure 14**. The results clearly demonstrate that YOLOv11-MSDFF-RiceD outperforms YOLOv11n significantly, with our proposed model achieving higher detection accuracy and eliminating missed detections. The missed detections observed with YOLOv11 in some cases can be attributed to two primary factors. Firstly, the network structure of YOLOv11 has inherent limitations in feature extraction. It fails to fully capture the feature information that is truly useful for disease detection, leading the model to learn incorrect feature patterns and thereby compromising detection accuracy. Secondly, the complex farmland environment poses significant challenges. The model is exposed to a large amount of redundant and complex interference information during the learning process, making it difficult to accurately extract the effective features of the disease. This results in suboptimal detection performance. In contrast, YOLOv11-MSDFF-RiceD addresses these challenges through enhanced feature extraction capabilities and improved robustness to environmental complexities, ensuring more reliable and accurate disease detection.

**Figure 15:**
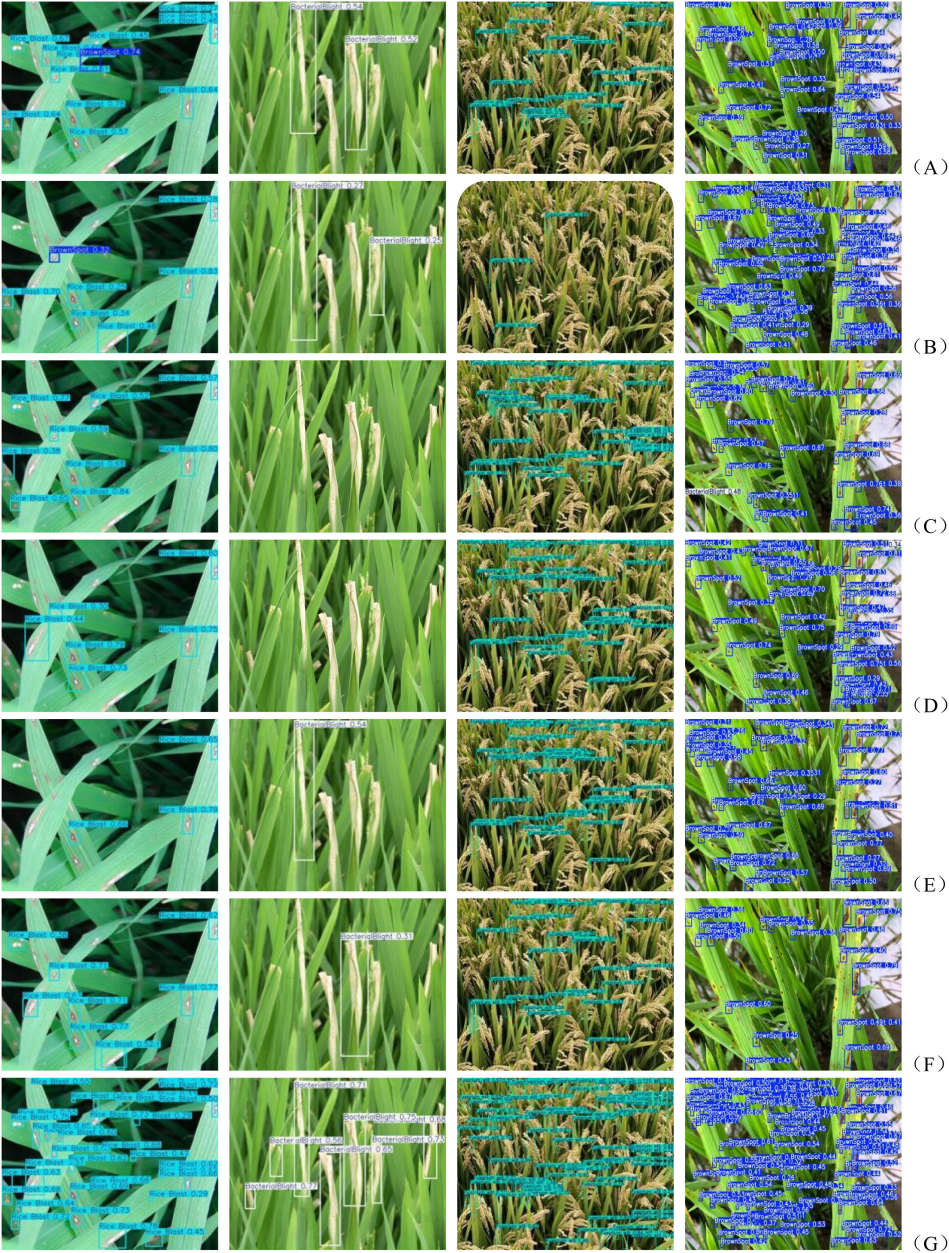
**(A-G)** Comparison of the effects of different models.

The comparison of the effects in **Figure 15** shows the superiority of the YOLOv11-MSDFF-RiceD model over other models such as YOLOv5, YOLOv6, YOLOv8, YOLOv9, YOLOv10 and YOLOv11 in rice disease detection tasks. From the results, in addition to YOLOv11-MSDFF-RiceD, other models generally have missed detection during the detection process, and YOLOv5 and YOLOv6 have the problem of misidentification of rice blast as brown spot. These missed and false detections not only affect the accuracy of disease detection, but also may mislead the actual disease management. The YOLOv11-MSDFF-RiceD model significantly reduces the missed detection and false detection, improves the detection accuracy, and can more accurately identify rice diseases including rice blast and brown spot. Although the model achieves good detection performance (89.8% mAP @ 0.5), its accuracy will decrease under extreme background or low resolution input. Similarly, small lesions (< 10 pixels) in severely occluded areas also showed a high false negative rate. Future work will explore a hybrid architecture that combines attention mechanisms with super-resolution preprocessing to address these challenges. In addition, although in this study, there was no misjudgment between diseases in the model, this does not mean that similar problems will not occur in subsequent studies, which also sounded the alarm for us. In order to prevent the occurrence of such problems, future research will focus on the following two aspects: First, expand the scale of the data set, especially increase the number of disease samples with similar symptoms, so as to enhance the adaptability of the model to complex situations; the second is to continuously optimize the feature extraction method to further improve the model ‘s ability to capture subtle differences, so as to better achieve the goal of accurate classification.

### 3.7 Model deployment comparison experiment

In this study, in order to highlight the performance advantages of lightweight models, we deployed multiple models on the Jetson Orin Nano development board and compared their frame rates. TensorRT is not used for acceleration processing during deployment. **Table 8** shows the frame rate differences between different models in detail. This comparison is mainly based on the video stream data taken by the drone. The flight parameters of the drone are: the flight speed is 3 m/s to 5 m/s, and the flight height is 3 m to 4 m away from the rice plant.

**Table 8:** Comparison of deployment speed of different models.

Modules	FPS	Preprocess
YOLOv5n	15	156ms
YOLOv6n	10	263ms
YOLOv8n	12	189ms
YOLOv9t	9	298ms
YOLOv10n	12	178ms
YOLOv11n	15	147ms
YOLOv11-MSDFF-RiceD	27	112ms

It can be seen from the results that the YOLOv11-MSDFF-RiceD model shows significant real-time and deployable advantages on the Jetson Orin Nano development board. The frame rate is as high as 27 FPS, and the preprocessing time is only 112 ms, which are significantly better than other models. This performance enables it to complete target detection quickly and efficiently in farmland disease detection tasks, and is suitable for real-time deployment in a resource-constrained hardware environment (Li et al., 2025). It provides a strong theoretical basis for the subsequent deployment of hardware equipment to drones, and provides strong support for rapid monitoring and precise prevention and control of farmland diseases.

## 4 Conclusion

Aiming at the challenge of rice leaf disease detection in complex field environment, this study proposes a lightweight network model based on improved multi-scale dynamic feature fusion based on YOLOv11 framework, named YOLOV11-MSDFF-RiceD. The model introduces the concept of ParameterNet, and replaces the original neck feature extraction network by designing the FlexiC3k2Net module to enhance the model ‘s ability to learn features and control the increase in computation. In addition, this study designs an efficient multi-scale feature fusion module (Hyper Multi-Scale Fusion Module, Hyper-MFFM), which aims to improve the computational efficiency and feature capture ability of the model, while reducing the number of parameters and memory usage. In terms of loss function, this study uses the auxiliary bounding box and the scale factor bounding box regression loss function (inner-WIoU) to improve the prediction accuracy of the model. Finally, through the Dependency Graph (DepGraph) pruning technique, the model volume is reduced and the computational load is reduced at a moderate sacrifice of model accuracy.

The experimental results show that the YOLOv11-MSDFF-RiceD model significantly reduces the computational load and model size (4.7 MB) while maintaining considerable detection accuracy. Although the improvement on mAP @ 0.5 is modest (1.7%), the lightweight design of the model addresses the urgent need to deploy AI solutions on edge devices with limited computing resources, such as drones or handheld agricultural sensors. Although the model shows robustness in complex farmland environments, there are still some challenges. Firstly, the dataset mainly covers four common rice diseases, and its performance in rare or emerging disease categories has not been tested. Secondly, changes in light conditions (such as overexposure or shadows) and background interference (such as overlapping leaves or soil patterns) may reduce the detection reliability. For example, under weak light conditions, the thin strip lesions of bacterial blight may be confused with natural veins. In addition, due to the limitation of rice cycle, this study did not deeply explore the influence of different heights and flight speeds on model training and detection performance during data acquisition. Future research will focus on expanding the data set to include more disease types and environmental changes, integrating illumination invariant feature extraction techniques to enhance robustness, and planning to study the effects of different altitudes and flight speeds on model performance. At the same time, future research directions also include optimizing the model structure and parameters to improve its robustness in complex scenarios, and exploring advanced technologies such as transfer learning and federated learning to further improve the performance of the model on embedded devices and ensure its effective deployment and application in actual agricultural scenarios.

## Data Availability

The original contributions presented in the study are included in the article/supplementary material. Further inquiries can be directed to the corresponding authors.
